# Plasma versus Serum Analysis by FTIR Spectroscopy to Capture the Human Physiological State

**DOI:** 10.3390/biotech11040056

**Published:** 2022-12-09

**Authors:** Rúben Araújo, Luís Ramalhete, Edna Ribeiro, Cecília Calado

**Affiliations:** 1 NMS—NOVA Medical School, Campo dos Mártires da Pátria 130, 1169-056 Lisboa, Portugal; 2 CHRC—Comprehensive Health Research Centre, Rua Câmara Pestana 6, 1150-199 Lisboa, Portugal; 3 IPST—Instituto Português do Sangue e da Transplantação, Alameda das Linhas de Torres—nr.117, 1769-001 Lisboa, Portugal; 4 H&TRC—Health & Technology Research Center, ESTeSL—Escola Superior de Tecnologia da Saúde, Instituto Politécnico de Lisboa, Avenida D. João II, lote 4.69.01, Parque das Nações, 1990-096 Lisboa, Portugal; 5 CIMOSM—Centro de Investigação em Modelação e Optimização de Sistemas Multifuncionais, ISEL—Instituto Superior de Engenharia de Lisboa, Instituto Politécnico de Lisboa, Rua Conselheiro Emídio Navarro 1, 1959-007 Lisboa, Portugal

**Keywords:** epigallocatechin-3-gallate, FTIR spectroscopy, plasma, serum

## Abstract

Fourier Transform InfraRed spectroscopy of serum and plasma has been highly explored for medical diagnosis, due to its general simplicity, and high sensitivity and specificity. To evaluate the plasma and serum molecular fingerprint, as obtained by FTIR spectroscopy, to acquire the system metabolic state, serum and plasma spectra were compared to characterize the metabolic state of 30 human volunteers, between 90 days consumption of green tea extract rich in Epigallocatechin-3-gallate (EGCG). Both plasma and serum spectra enabled the high impact of EGCG consumption on the biofluid spectra to be observed, as analyzed by the spectra principal component analysis, hierarchical-cluster analysis, and univariate data analysis. Plasma spectra resulted in the prediction of EGCG consumption with a slightly higher specificity, accuracy, and precision, also pointing to a higher number of significant spectral bands that were different between the 90 days period. Despite this, the lipid regions of the serum spectra were more affected by EGCG consumption than the corresponding plasma spectra. Therefore, in general, if no specific compound analysis is highlighted, plasma is in general the advised biofluid to capture by FTIR spectroscopy the general metabolic state. If the lipid content of the biofluid is relevant, serum spectra could present some advantages over plasma spectra.

## 1. Introduction

Fourier Transform Infra-Red (FTIR) spectroscopy has been extensively explored in the last decade in a myriad of biomedicine applications, specifically in the mid-infrared region of the spectra (between 4000 and 400 cm^−1^), as this region covers the molecular fundamental vibrations of a high diversity of functional groups in complex biological samples, such as CC, C=C, CO, C=O, CN, CP, CH, PO, CP, OH, and NH. Examples of applications include metabolite quantification [1], monitoring of stem cell differentiation [2], transfection events [3], infection processes [4,5], medical diagnosis, prognosis, and therapy monitoring [6,7].

Major advantages of FTIR spectroscopy pertain to its simplicity, speed, and economic benefits. Economic, for no expensive reagents are needed; simplicity, since it usually only requires simple sample pre-processing (e.g., a dehydration step) or even no pre-processing at all; and it is rapid to apply, as a spectrum is usually acquired in 1 min. Furthermore, the technique is flexible as it enables the processing of highly different types of biological samples, e.g., culture media, tissues, cells, and biofluids, taking advantage of diverse modes of detection such as transmission, reflectance, and attenuated total reflection. The analysis can be conducted in high-throughput reading systems, including plates with multi-micro wells, e.g., with 384 wells for 5 µL samples, or through fiber optical cables, providing in situ real time analysis, or to a microscope that, if associated to a focal plane arrays detector, e.g., with 64 x 64 array detector, enables 2D analysis and detection of residual components [8].

With the goal of enabling a medical diagnosis, based on minimal invasive analysis, FTIR spectroscopy has been applied to diverse human biofluids, such as blood, saliva, urine, semen, vaginal fluid, tears and sweat [9,10,11,12,13]. Biofluid analysis presents advantages such as relatively easy processing, clinical availability, and its presence in diverse biobanks. From all body fluids, serum and plasma have been the most analyzed, as they allow the overall metabolic state of the organism to be acquired. Examples of the analysis of these biofluids by these techniques for medical diagnosis include blood-related diseases as leukemias and other cancers [10], rheumatoid arthritis [14], diagnosis and differentiation of multiple sclerosis and amyotrophic lateral sclerosis [15] mental disorders as bipolar and schizophrenia [16], and dementia [17].

Usually, the blood cell-free components such as plasma and serum are analyzed. Either plasma or serum may be preserved with a simple freezing procedure, contrary to whole blood, as in this last biofluid, freezing would result in cell disruption. With erythrocytes being the main cell blood component, the FTIR spectra of frozen blood would mainly reflect the erythrocyte content, i.e., hemoglobin, which would mask the spectra of other molecules. This list is not comprehensive but, in general, there are indications as to which biofluid should be used for a specific clinical analysis [18,19]. Serum is used for diverse immunological analysis such as complement fixation agglutination tests, whereas, for heterogeneous immunoassays and due to potential interference by fibrinogen, plasma is advised. For other assays such as hemagglutination tests, ELISAs, or immunoblots, generally, either serum or plasma can be used [20]. Plasma is advised to analyze lipoproteins or apolipoproteins, since these molecules are mostly found in the plasma fraction after low-speed centrifugation [21], most probably due to the elimination of these molecules with the clot [22]. The coagulation process can lead to the cell’s secretion of diverse factors, resulting in the different composition of metabolites between serum and plasma, as with vascular endothelial growth factor, which result in these metabolites being up to eight times more concentrated in serum than in plasma [23]. Plasma is chosen when there is a need to evaluate matrix metalloproteinases and tissue inhibitor of matrix metalloproteinase, for clinical and diagnostic purposes, especially in vascular biology and atherothrombotic syndromes, as platelets and leukocytes that contain these molecules could release them during the coagulation cascade [24]. Plasma is also advised when studying blood microvesicules to minimize the capture of microvesicules by the blood clot, for the microvesicules hydrolysis by the proteases activated by the coagulation cascade, or even due to the creation of platelet microvesicules, a result from platelet activation [25]. Since FTIR spectroscopy retrieves the overall molecular composition of the system, there is no indication of the best biofluid to analyze in order to predict a specific disease.

Due to the potential of FTIR spectroscopy to predict physiological states, the present works aims to compare FTIR spectroscopic analysis of serum and plasma to predict a specific human metabolic state. Plasma and serum samples taken from 30 human volunteers, before and after 90 days consumption of green-tea extracts rich in epigallocatechin-3-gallate (EGCG) was considered. EGCG is the most abundant catechin in tea, representing between 50% and 80%, has been associated with antioxidant and free radical scavenging properties [26,27], and has been observed to have a positive impact in health against a range of human-ailing conditions such as cancer, cardiovascular diseases, diabetes, obesity, inflammatory diseases, and infections [28]. In a previous work, it was observed that 90 days consumption of EGCG resulted in a significant impact (*p* < 0.05) of blood erythrocytes, hemoglobin, hematocrit, mean cell volume, reticulocyte hemoglobin content, fetal hemoglobin level, and the ratio between Low-Density and High-Density Lipoproteins (LDL/HDL) [29]. A significant alteration of the plasma spectra after EGCG consumption was also observed. In the present work, plasma and serum spectra are compared to predict the physiological state based on same 30 human volunteers before and after the 90 days of EGCG consumption.

## 2. Materials and Methods

### 2.1. Study Population and Design

This was an interventional, uncontrolled, prospective, longitudinal, and individual analysis study, which included 30 healthy individuals (with no previously diagnosed pathologies). Inclusion criteria considered were adult voluntaries (ages superior to 18 years old and less than 65 years old) with no acknowledged previously diagnosed pathology of any type. Exclusion criteria applied included viral infections, consumption of tea, and forgotten capsules on consecutive days during the study. Data were analyzed under blind conditions.

### 2.2. Collection of Biological Samples

Peripheral blood was collected in a tube with anticoagulant ethylenediaminetetraacetic acid (EDTA) VACUETTE^®^ for plasma analysis and in a serum tube with no anticoagulant VACUETTE^®^, using standard blood collection procedures at time 0 (T0) and after 90 days (T90) of the daily ingestion of 225 mg EGCG/capsule [29]. Serum and plasma samples were refrigerated and maintained at 4 °C until blood centrifugation. Serum and plasma were obtained by centrifugation at 3500 rpm for 10 min (Mikro 220T, Hettich, Tuttlingen, Germany) and maintained at −20 °C until FTIR spectra acquisition.

### 2.3. FTIR Spectra Acquisition

A transmission detection mode associated to a high-throughput reading system with 96-well plates was used [3,4,30] was used. Briefly: triplicates of 25 μL of plasma and serum, diluted at 1/10 in water, from each volunteer at both T0 and T90, were transferred to a 96-wells Si plate and then dehydrated for about 2.5 h, in a desiccator under vacuum (Vacuubrand, ME 2, Wertheim, Germany). Spectral data was collected using a FTIR spectrometer (Vertex 70, Bruker, Billerica, MA, USA) equipped with an HTS-XT (Bruker) accessory. Each spectrum represented 64 coadded scans, with a 2 cm^−1^ resolution, and was collected in transmission mode, between 400 and 4000 cm^−1^. The first well of the 96-wells microplate did not contain a sample and the corresponding spectra was acquired and used as background, according to the HTS-XT manufacturer.

### 2.4. Spectra Preprocessing and Processing

Spectra were preprocessed by atmospheric and baseline correction, and second derivative using a Savitzky–Golay filter, with a 2nd order polynomial over a 15-point window with unit vector normalization. Atmospheric correction was conducted with OPUS^®^ software, version 6.5 (Bruker, Bremen, Germany; Billerica, MA, USA), whereas the second derivative, unit vector normalization and processing methods, such as principal component analysis (PCA) and hierarchical cluster analysis (HCA) were conducted with The Unscrambler^®^ X, version 10.5 (CAMO software AS, version 10.4, Oslo, Norway). HCA was based on Spearman’s rank correction (distance measure) and hierarchical average-linkage (clustering method). The t-student analysis of spectral bands between T0 and T90 was performed on Microsoft Excel™.

## 3. Results

**Figure 1:**
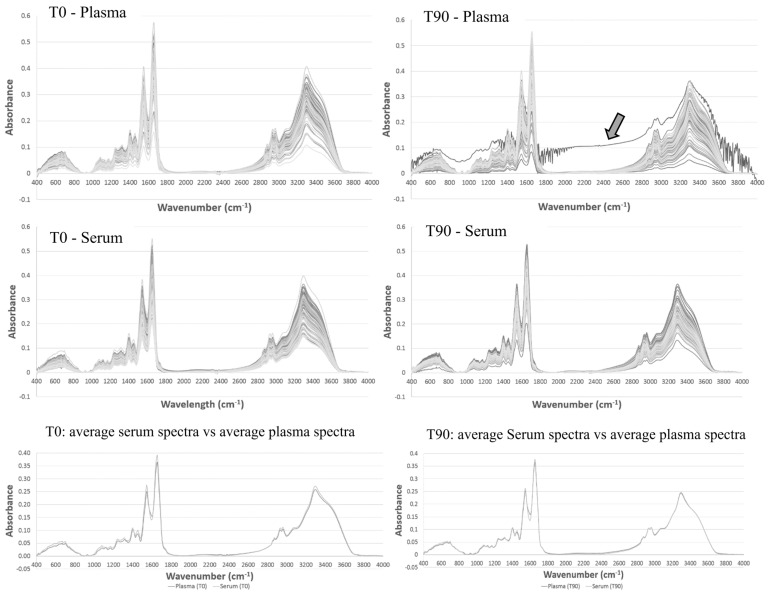
Plasma and serum spectra after atmospheric and baseline correction obtained at T0 and T90, representing all triplicated analysis of the 30 volunteers or in alternative its mean spectra. The arrow indicates the unique spectra eliminated from the subsequent studies.

All biological samples (i.e., plasma and serum) were processed by the same general protocol, i.e., all the blood samples were maintained 2 days at 4 °C before centrifugation, and the plasma and serum obtained were kept frozen at −20 °C for several weeks before spectra acquisition. Serum and plasma samples were previously diluted in water before spectra acquisition. For each sample, triplicate dilutions were conducted. Figure 1 represents the spectra obtained from all the analyzed samples (serum and plasma) before the EGCG consumption (T0) and after the 90 days of EGCG consumption (T90), preprocessed with atmospheric and baseline correction. From all the samples analyzed by FTIR spectroscopy (*n* = 360, corresponding to the triplicates of plasma and serum samples of 30 participants acquired at the two-time intervals, T0 and T90), only one spectrum (corresponding to one replica of triplicate measurements) was eliminated, and not considered for subsequent analysis. This spectrum, indicated by an arrow in Figure 1, presented a very low signal to noise ratio and reflected most probably a human error.

It was observed that serum and plasma spectra, obtained from different volunteers, led to spectra with different absorbance values, pointing to the FTIR spectroscopy sensibility to capture the differences between the biofluid composition among the 30 volunteers (Figure 1). The highest absorbance value of the whole spectra was the 1656 cm^−1^ band, associated with amide I vibrations on proteins of plasma and serum samples. This band, of all plasma samples (i.e., T0 and T90 together), was, in mean terms, significantly different from the band of all the serum samples (*p* = 0.06) (Table 1). The band of plasma T0 samples was also statistically different from T0 serum samples (*p* = 0.04). The following groups of this band were not statistically different: plasma T0 samples versus plasma T90 samples; serum T0 samples versus serum T90 samples; and plasma T90 versus serum T90 samples (Table 1).

**Figure 2:**
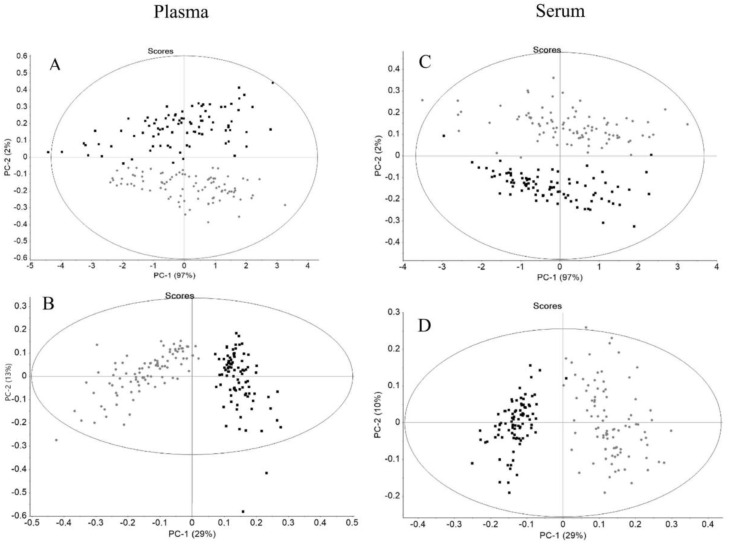
PCA of plasma (**A**,**B**) and serum (**C**,**D**) with spectra pre-processed by atmospheric and baseline correction (**A**,**C**) and second derivative and unit vector normalization (**B**,**D**) relative to T0 (grey symbols) and T90 (black symbols), respectively, with Hotelling’s T^2^ ellipse at a 99% confidence interval.

In the PCA score-plot of serum and plasma spectra, the different volunteers were represented in different positions, either before and after EGCG consumption (Figure 2), indicating that the spectra could pick up the different molecular compositions of serum and plasma samples. Furthermore, the serum and plasma spectra captured the effect of EGCG consumption, since plasma and serum samples, after EGCG consumption, i.e., the T90 samples, were in distinct clusters comparatively to the T0 samples (Figure 2).

For both plasma and serum spectra, T0 data were separated in the PCA score-plot in relation to T90 data after atmospheric and baseline correction (Figure 2A,C). Sample separation between T0 and T90 was more evident with normalized second derivative spectra, as expected (Figure 2B,D), since the second derivative resolved superimposed peaks. Spectra normalization was also performed, unlike the previous conducted work by the authors [29], in order to minimize the effect of confounding factors such as varying thickness of sample while highlighting the differences in the sample biochemical constituents [8].

The PCA score plot presents the Hotelling’s T2 ellipse with a 99% confidence interval, with samples outside of the same representing possible outliers (Figure 2). In both biofluids, only one replica of the triplicate measurement of the 30 volunteers could be considered as an outlier. Despite this, in the following analysis, these two samples were not removed.

**Figure 3:**
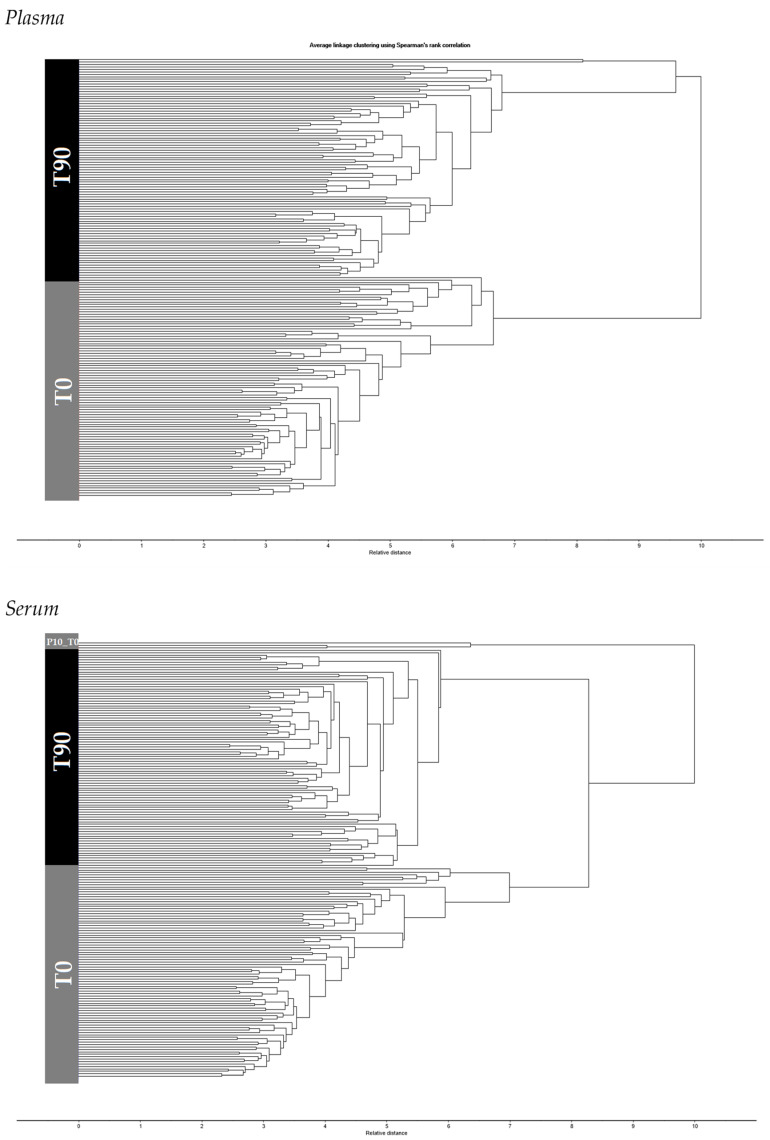
HCA of plasma and serum spectra pre-processed by atmospheric correction, second derivative, and unit vector normalization, relative to T0 and T90, respectively.

In general, it was possible to differentiate, in a dendrogram, T0 from T90 samples, based on the normalized second derivative spectra of plasma and serum samples, respectively (Figure 3). In the HCA, all plasma samples were correctly classified as belonging to T0 or T90 group, again highlighting the FTIR spectroscopy sensibility to capture the differences between before and after the biocompound consumption. Concerning serum, only samples from one volunteer taken before EGCG consumption (i.e., at T0) were misclassified as belonging to the T90 group. Therefore, serum presented itself, when compared to plasma, with a slightly lower specificity (0.97), precision (0.97),and accuracy (0.98) in its ability to predict if a given volunteer had consumed EGCG.

**Figure 4:**
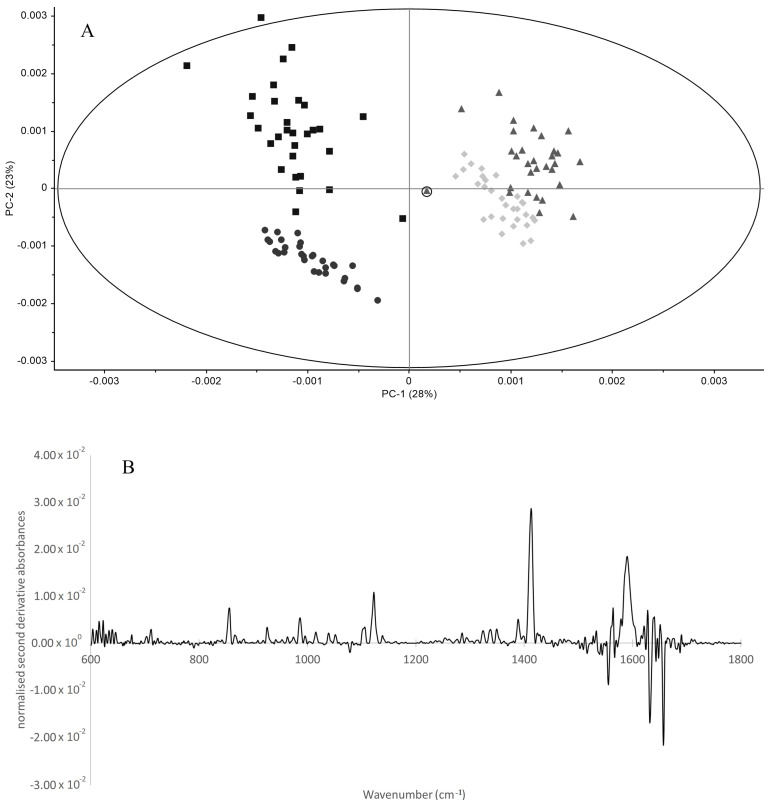
PCA of normalized second derivative spectra, between 600 and 3000 cm^−1^, for plasma at T0 (squares) and T90 (circles), and serum samples at T0 (triangles) and T90 (diamond), with Hotelling’s ellipse at 1% significance (**A**); and the corresponding PC1 loading vector (**B**). The sample in “A” marked as a triangle with an outside circle represents the unique sample misclassified in the HCA (corresponding to a serum sample at T0 from volunteer 10).

The PCA represented in Figure 4A includes both serum and plasma spectra after normalization and second derivative. The spectra regions between 600 and 3000 cm^−1^, instead of the whole spectrum (i.e., between 400 and 4000 cm^−1^) were considered in order to minimize the influence of noise amplification at the spectra limits. The placement of serum and plasma samples on different quadrants of the score-plot reflects the serum different molecular composition in relation to plasma samples. One unique sample was classified as an outlier, considering the Hotelling’s ellipse at 1% (Figure 4A), pertaining to a plasma sample at T0, of volunteer number 27. Despite this, the sample was well classified in HCA (Figure 3). The unique sample that was not well classified in both HCA (i.e., with whole or partial spectra), was a serum sample at T0 from volunteer 10, marked by a circle in Figure 4A. This sample is closer to the samples T90 cluster than the T0 clusters in the PCA score-plot (Figure 4A). In the HCA based on the whole spectra (Figure 3), this serum sample was wrongly classified in the T90 cluster, where in the HCA based on the 600 to 3000 cm^−1^ spectral range, it was classified in a third cluster, besides the two clusters with T0 and T90 samples (data not shown). Interestingly, this volunteer presented the lowest hemoglobin content at T0 in relation to all 30 participants, with a value approximately 20% lower than the mean of the other volunteers at T0. After EGCG consumption, the hemoglobin level of this volunteer was not significantly different from other participants. All this points to the serum spectra as being more sensitive to capture the system metabolic state.

The loading vector of PC1, represented in Figure 4B, points to the spectral regions that most contributed to plasma and serum samples separation on the score-plot, and consequently, the spectral bands that were the most different between these types of samples. The following major regions were identified as discriminating plasma and serum: 1656, 1632, 1555, and 1590 cm^−1^ associated with amide II and I of proteins, 1412 cm^−1^ associated most probably with CH3 bands in lipids, and 1122 cm^−1^ associated with carbonyl vibrations of carbohydrates and the 855 cm^−1^ band of the fingerprint region.

A univariate data analysis of bands from the normalized second derivative spectra of plasma and serum was subsequently conducted (Table 2 and Table 3). To minimize the effect of noise amplification (highlighted by the second derivative), only shared (negative) bands present in samples of six different volunteers, randomly selected, were considered. Based on this, a total of 19 and 29 negative bands were identified on serum and plasma normalized second derivative spectra, respectively. Based on a paired Student’s *t*-test, 13 and 23 of these bands were statistically different (*p* < 0.05) between T0 and T90 from serum and plasma samples, respectively (Table 2 and Table 3). This points to plasma spectra being richer in information concerning the biofluid molecular composition in relation to the serum spectra, and in capturing the effect of EGCG consumption. Despite this, the following spectral bands of serum samples showed a more significative change after EGCG consumption in relation to plasma samples: asymmetric vibrations on the CH3 (2960 cm^−1^) and CH3 (2926 cm^−1^) groups and symmetric vibration of CH2 (2853 cm^−1^) in lipids, and asymmetric vibrations of phosphate bonds (1240 cm^−1^).

**Figure 5:**
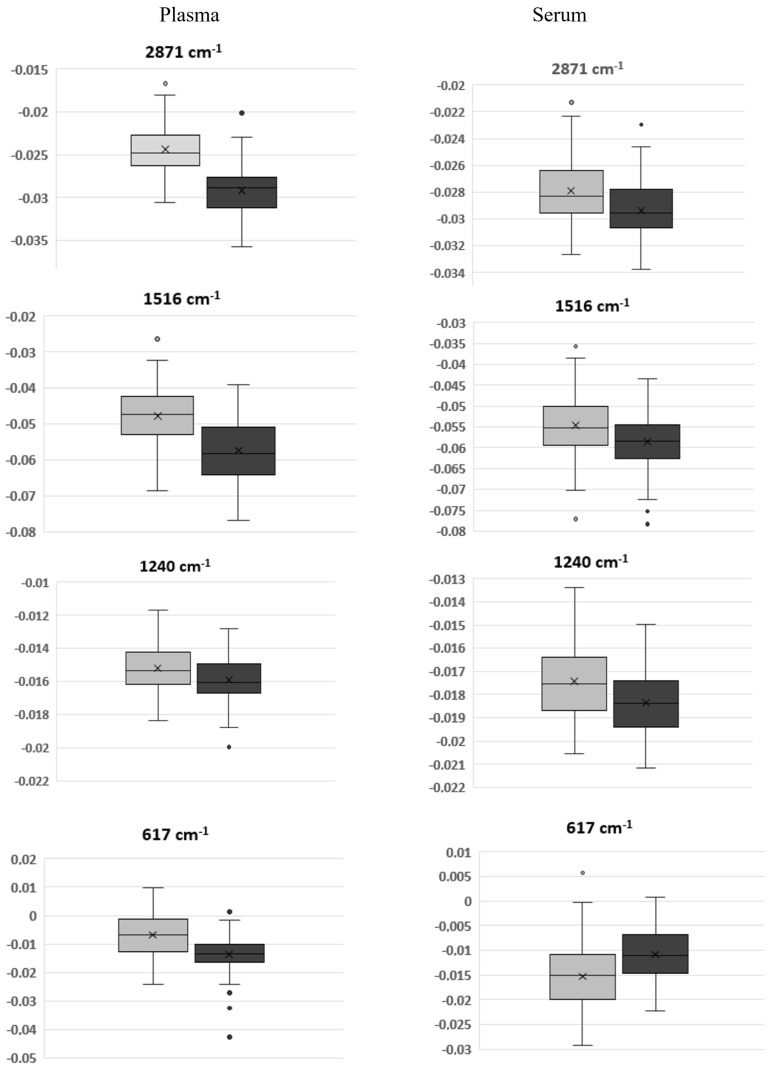
Boxplots of some negative bands of the normalized second derivative spectra of plasma (left side graphs) and serum (right side graphs). Grey and black panels display T0 and T90 samples, respectively.

The following 10 common bands were significantly different between T0 and T90 for both plasma and serum spectra: 2871, 1455 and 1400 cm^−1^ due to CH2 and CH3 vibrations in lipids, 1656 and 1516 cm^−1^ due to amide II and I, respectively from proteins, 1240 and 1081 cm^−1^ due to phosphate groups in diverse molecules as phospholipoids, 1032 cm^−1^, due to carboxyl groups as in glucose or other carbohydrates and 700 and 617 cm^−1^ on the fingerprint regions from diverse molecules (Figure 5, Table 2 and Table 3).

## 4. Discussion

FTIR spectroscopic analysis of serum and plasma has been extensively evaluated for medical diagnostics due to its easy access and since its molecular composition represents diverse pathophysiological states. However, to this date and to the best of our knowledge, there is no study comparing plasma and serum, to retrieve the whole molecular fingerprint by FTIR spectroscopy, to evaluate human physiological states. In the present work, plasma and serum spectra were compared to predict the physiological state based on plasma and serum analysis of 30 human volunteers after 90 days of EGCG consumption, according to the impact previously observed on diverse blood constituents, as erythrocytes, hemoglobin, hematocrit, mean cell volume, reticulocyte hemoglobin content, fetal hemoglobin level, and the ratio between Low-Density and High-Density Lipoproteins (LDL/HDL) [29].

It was observed that the amide I band of serum samples (including T0 and T90 or T0 alone) presented significantly higher absorbances than the corresponding plasma samples (Table 1). The higher absorbance values of this band, corresponding to protein content of serum samples, were not in accordance with serum, theoretically, presenting a lower protein content due to the elimination of the blood clot. It was expected that serum and plasma would present similar protein concentrations as the major protein constituents in blood are proteins, such as albumin and immunoglobulins, which are not significantly affected by the blood clot removal. Despite this, due to fibrinogen removal, it was expected that serum would have slightly lower protein concentration. For example, Lum and Gambino [31] detected protein mean values of 7.21 g/L in serum and 7.45 g/L in heparinized plasma. The higher amide I band observed in serum could have resulted from favoring peptide and proteins secretion, among other molecules by the blood clot [31,32], since blood samples were maintained for 2 days before centrifugation. The higher amide I band in serum in relation to plasma could also result from protein denaturation [33], since serum and plasma samples were maintained frozen at −20 °C for various weeks before spectra acquisition, therefore leading to the hypothesis that protein denaturation could occur in higher percentage in serum samples. Another possibility to explain the higher amide I absorbance of serum samples is the effect of the anticoagulant leading to osmotic alterations due to the redistribution of fluid between blood cells and plasma, that may result in plasma dilution, consequently leading to some molecules lower concentrations. For example, Ayache et al. [34] comparing soluble factors, observed that plasma presented only fibrinogen and osteopontin in higher concentrations in relation to serum, in which serum presented 18 factors in higher quantities than in plasma, including 11 chemokines. A higher concentration in the serum of albumin concentration (4.41 g/L) than in plasma (4.29 g/L), as well as other components as alkaline phosphatase, phosphorous, triglyceride, and uric acid was also registered [31]. Moreover, from 72 common metabolites present in serum and plasma, as analyzed by gas chromatography/time-of-flight mass spectrometry, 36 (50%) discriminated serum from plasma, whereas 29 were present in higher concentrations in serum [32].

In the present work, it was observed that FTIR spectroscopy enabled, serum to be discriminated from plasma composition, with a high sensitivity and specificity, as pointed out by the PCA of normalized second derivative spectra (Figure 4). It was also observed that both plasma and serum spectra enabled predictions, with a high sensitivity and specificity, of the volunteers’ physiological state since it was possible to discriminate from both the spectra of the biofluids, the volunteers before and after a specific biocompound consumption. This was observed by an analysis of isolated bands, i.e., the bands univariate data analysis (Table 2 and Table 3) and by multivariate data analysis by PCA (Figure 2 and Figure 4) and HCA (Figure 3) of the whole or partial spectra. For example, with both biofluids, it was possible by the use of HCA to predict if a biofluid sample was obtained after the 90 days period with 100% sensitivity and specificity, precision, and accuracy higher than 0.97. This was in accordance with the 90 days impact observed on diverse blood parameters [29].

It was also observed that plasma is, apparently, a richer biofluid regarding the molecular information acquired by FTIR spectroscopy, since a higher number of shared spectral bands of the normalized second derivative spectra were found among the volunteers, comparatively to serum, of 29 and 19 on plasma and serum spectra, respectively. From these bands, a 1.8-fold-higher number of plasma bands (*n* = 23) were significantly different between T0 and T90, comparatively to serum bands (*n* = 13) (Table 2 and Table 3). The plasma as a richer biofluid to retrieve spectral information was also corroborated by the higher dispersion of plasma samples on the score-plot of the spectra PCA in relation to serum samples (Figure 4A). A higher impact of 90 days interval in data dispersion on the PCA score-plot pertaining to plasma samples was also observed when compared to serum samples, i.e., it was observed from the plasma spectra a higher decrease in samples dispersion on the PCA score-plot at T90 in relation to T0, when compared to serum samples.

Despite this, a higher sensitivity of the serum spectra was also observed in predicting a different molecular signature for one patient at T0, that presented the lowest hemoglobin concentration from all 60 samples. Consequently, the HCA of serum spectra, resulted in a classification of patients at T90 with slightly lower specificity (97%), accuracy (98%) and precision (97%), in relation to plasma spectra. Furthermore, there were serum spectral bands that were more significantly affected by the 90 days interval than in plasma spectra, such as asymmetric vibrations on the CH3 (2960 cm^−1^) and CH3 (2926 cm^−1^) groups and symmetric vibration of CH2 (2853 cm^−1^) from lipids, and asymmetric vibrations of phosphate bonds (1240 cm^−1^). Interestingly, the spectral region between 2853 and 2960 cm^−1^ focuses on the lipids regions and are according to the impact of EGCG consumption on the blood LDL/HDL [29]. The lipids regions highlighted in serum spectra, in relation to plasma spectra, could also be associated to the impact of the clotting process in increased lipids as pointed out, e.g., by Ishikawa et al. (2014) [35], that in a lipidomic based work, observed higher concentrations of diverse lipids in serum in relation to plasma, that could have resulted from the clotting process, leading the researchers to advise plasma as optimal matrix for exploring lipid biomarkers.

## 5. Conclusions

Briefly, the spectra of both biofluids, i.e., plasma and serum, enabled, in general, the acquisition of the molecular fingerprint associated to different physiological states with high sensitivity, specificity, accuracy and precision. Despite this, plasma was a richer biofluid regarding the molecular information captured by the spectra. Therefore, in those cases where there is no specification towards a specific analysis, plasma would be the advisable biofluid to be analyzed. There can be cases where serum could present advantages in the identification of defined physiological states, e.g., in the present study, where it enabled to highlight the patient with the lowest blood hemoglobin concentration. Another advantage of using plasma is to minimize the effect of the clotting process, especially when blood centrifugation is not quickly conducted after blood collection.

## Figures and Tables

**Table 1:** Absorbance at 1656 cm^−1^ (amide I). The mean value and standard deviation are presented. The p-value of a Student’s *t*-test analysis is also present.

Absorbance at 1656 cm^−1^			Student’s *t*-Test	
	Mean	Standard Deviation	Analyzed Group	*p*-Value
Plasma T0	0.36	0.07	Plasma T0 vs. Plasma T90	>0.1
Plasma T90	0.36	0.07	Serum T0 vs. Serum T90	>0.1
Serum T0	0.39	0.06	Plasma (T0 + T90) vs. Serum (T0 + T90)	0.06
Serum T90	0.37	0.05	Plasma T0 vs. Serum T0	0.04
			Plasma T90 vs. Serum T90	>0.1

**Table 2:** Mean values and standard deviations of negative bands of the normalized second derivative spectra of plasma at T0 and T90, respectively, and *p*-value of Student’s *t*-test regarding the comparison between T0 and T90, at *p* < 0.05.

Wavenumber (cm^−1^)	T0		T90		*p*-Value (T0 vs. T90)
	Mean	Standard Deviation	Mean	Standard Deviation	
2872	−2.50 × 10^−2^	2.88 × 10^−3^	−3.06 × 10^−2^	2.53 × 10^−3^	7.14 × 10^−22^
1738	1.06 × 10^−3^	7.62 × 10^−3^	−8.46 × 10^−3^	3.40 × 10^−3^	3.5 × 10^−20^
1690	−2.95 × 10^−2^	6.79 × 10^−3^	−3.59 × 10^−2^	6.81 × 10^−3^	3.47 × 10^−9^
1656	−1.55 × 10^−1^	1.98 × 10^−2^	−1.49 × 10^−1^	1.86 × 10^−2^	3.39 × 10^−2^
1639	−8.74 × 10^−2^	2.05 × 10^−2^	−1.00 × 10^−1^	2.86 × 10^−2^	7.66 × 10^−4^
1545	−1.26 × 10^−1^	1.21 × 10^−2^	−1.17 × 10^−1^	1.37 × 10^−2^	9.68 × 10^−7^
1515	−4.22 × 10^−2^	7.04 × 10^−3^	−5.38 × 10^−2^	7.42 × 10^−3^	4.97 × 10^−17^
1469	−3.20 × 10^−2^	4.63 × 10^−3^	−3.56 × 10^−2^	5.05 × 10^−3^	9.97 × 10^−6^
1455	−4.71 × 10^−2^	6.06 × 10^−3^	−4.14 × 10^−2^	7.70 × 10^−3^	2.13 × 10^−7^
1440	−1.81 × 10^−2^	3.98 × 10^−3^	−2.17 × 10^−2^	3.18 × 10^−3^	5.32 × 10^−10^
1400	−4.54 × 10^−2^	4.00 × 10^−3^	−4.83 × 10^−2^	3.34 × 10^−3^	1.03 × 10^−7^
1340	−2.29 × 10^−2^	8.48 × 10^−3^	−1.04 × 10^−2^	2.17 × 10^−3^	8.05 × 10^−26^
1285	−6.35 × 10^−3^	1.30 × 10^−3^	−4.53 × 10^−3^	1.24 × 10^−3^	2.82 × 10^−13^
1240	−1.52 × 10^−2^	1.44 × 10^−3^	−1.59 × 10^−2^	1.35 × 10^−3^	2.58 × 10^−3^
1172	−2.36 × 10^−2^	2.75 × 10^−3^	−2.18 × 10^−2^	3.97 × 10^−3^	1.25 × 10^−3^
1085	−9.93 × 10^−3^	2.34 × 10^−3^	−1.36 × 10^−2^	2.08 × 10^−3^	4.01 × 10^−19^
1031	−1.13 × 10^−2^	2.06 × 10^−3^	−9.26 × 10^−3^	2.58 × 10^−3^	3.44 × 10^−7^
928	−8.43 × 10^−3^	1.92 × 10^−3^	−1.27 × 10^−2^	2.01 × 10^−3^	4.02 × 10^−22^
854	−1.36 × 10^−2^	2.70 × 10^−3^	−1.17 × 10^−2^	2.85 × 10^−3^	6.51 × 10^−5^
745	−1.28 × 10^−2^	2.10 × 10^−3^	−8.73 × 10^−3^	3.11 × 10^−3^	2.06 × 10^−17^
700	−2.82 × 10^−2^	2.53 × 10^−3^	−2.64 × 10^−2^	3.89 × 10^−3^	3.63 × 10^−04^
631	−1.12 × 10^−2^	4.57 × 10^−3^	−2.03 × 10^−2^	5.53 × 10^−3^	4.99 × 10^−21^
616	−2.10 × 10^−3^	5.56 × 10^−3^	−1.25 × 10^−2^	6.55 × 10^−3^	1.53 × 10^−22^

**Table 3:** Mean values and standard deviations of negative bands of the normalized second derivative spectra of sera at T0 and T90, respectively, and the *p*-value of Student’s *t*-test regarding the comparison between T0 and T90, at *p* < 0.05.

Wavenumber (cm^−1^)	T0		T90		*p*-Value (T0 vs. T90)
	Mean	Standard Deviation	Mean	Standard Deviation	
2960	−3.19 × 10^−2^	2.27 × 10^−3^	−3.49 × 10^−2^	1.93 × 10^−3^	2.09 × 10^−18^
2926	−2.54 × 10^−2^	5.15 × 10^−3^	−2.75 × 10^−2^	5.08 × 10^−3^	1.41 × 10^−6^
2871	−2.79 × 10−^2^	2.45 × 10^−3^	−2.94 × 10^−2^	2.14 × 10^−3^	6.07 × 10^−6^
2853	−2.87 × 10^−2^	6.84 × 10^−3^	−2.51 × 10^−2^	7.30 × 10^−3^	3.25 × 10^−10^
1657	−1.54 × 10^−1^	1.67 × 10^−2^	−1.48 × 10^−1^	1.54 × 10^−2^	1.19 × 10^−2^
1516	−5.47 × 10^−2^	7.50 × 10^−3^	−5.86 × 10^−2^	6.52 × 10^−3^	3.72 × 10^−4^
1455	−4.69 × 10^−2^	5.26 × 10^−3^	−5.42 × 10^−2^	4.07 × 10^−3^	1.95 × 10^−17^
1400	−5.12 × 10^−2^	3.39 × 10^−3^	−5.02 × 10^−2^	3.03 × 10^−3^	2.79 × 10^−2^
1240	−1.74 × 10^−2^	1.59 × 10^−3^	−1.84 × 10^−2^	1.28 × 10^−3^	9.36 × 10^−6^
1081	−1.79 × 10^−2^	2.78 × 10^−3^	−1.96 × 10^−2^	1.93 × 10^−3^	7.47 × 10^−7^
1032	−1.21 × 10^−2^	2.23 × 10^−3^	−1.29 × 10^−2^	1.88 × 10^−3^	4.43 × 10^−3^
700	−2.94 × 10^−2^	2.98 × 10^−3^	−3.28 × 10^−2^	3.28 × 10^−3^	7.81 × 10^−10^
617	−1.52 × 10^−2^	6.76 × 10^−3^	−1.08 × 10^−2^	4.99 × 10^−3^	8.50 × 10^−8^
